# DNA methylation and type 2 diabetes: a systematic review

**DOI:** 10.1186/s13148-024-01670-6

**Published:** 2024-05-16

**Authors:** Nikhil Nadiger, Jyothisha Kana Veed, Priyanka Chinya Nataraj, Arpita Mukhopadhyay

**Affiliations:** 1 Research Scholar, Manipal Academy of Higher Education, Manipal, India; 2 Division of Nutrition, St. John’s Research Institute, St. John’s Medical College, St Johns National Academy of Health Sciences, Sarjapura Road, Koramangala, Bangalore, 560034 India; 3 Present Address: Vedantu, Bangalore, India

**Keywords:** Type 2 diabetes, DNA methylation, Epigenome-wide association studies, Epigenetics

## Abstract

**Objective:**

DNA methylation influences gene expression and function in the pathophysiology of type 2 diabetes mellitus (T2DM). Mapping of T2DM-associated DNA methylation could aid early detection and/or therapeutic treatment options for diabetics.

**Design:**

A systematic literature search for associations between T2DM and DNA methylation was performed. Prospero registration ID: CRD42020140436.

**Methods:**

PubMed and ScienceDirect databases were searched (till October 19, 2023). Preferred Reporting Items for Systematic Reviews and Meta-Analyses (PRISMA) guidelines and New Castle Ottawa scale were used for reporting the selection and quality of the studies, respectively.

**Result:**

Thirty-two articles were selected. Four of 130 differentially methylated genes in blood, adipose, liver or pancreatic islets (*TXNIP*, *ABCG1*, *PPARGC1A*, *PTPRN2*) were reported in > 1 study. *TXNIP* was hypomethylated in diabetic blood across ethnicities. Gene enrichment analysis of the differentially methylated genes highlighted relevant disease pathways (T2DM, type 1 diabetes and adipocytokine signaling). Three prospective studies reported association of methylation in *IGFBP2*, *MSI2*, *FTO*, *TXNIP*, *SREBF1*, *PHOSPHO1*, *SOCS3* and *ABCG1* in blood at baseline with incident T2DM/hyperglycemia. Sex-specific differential methylation was reported only for *HOOK2* in visceral adipose tissue (female diabetics: hypermethylated, male diabetics: hypomethylated). Gene expression was inversely associated with methylation status in 8 studies, in genes including *ABCG1* (blood), *S100A4* (adipose tissue), *PER2* (pancreatic islets), *PDGFA* (liver) and *PPARGC1A* (skeletal muscle).

**Conclusion:**

This review summarizes available evidence for using DNA methylation patterns to unravel T2DM pathophysiology. Further validation studies in diverse populations will set the stage for utilizing this knowledge for identifying early diagnostic markers and novel druggable pathways.

**Supplementary Information:**

The online version contains supplementary material available at 10.1186/s13148-024-01670-6.

## Introduction

Type 2 diabetes mellitus (T2DM) is a disorder of genetic and environmental factors. It is projected to affect 693 million people worldwide by 2045 [1]. DNA methylation had been proposed as one of the epigenetic phenomena for explaining the missing heritability of T2DM, as multiple, large genome-wide association studies have been able to account for only < 20% of the estimated T2DM heritability [2]. DNA methylation is an epigenetic phenomenon in which the C5 carbon of the cytosine residue is attached to a methyl group, predominantly in cytosine-phosphate-guanine (CpG) sites [3–5]. This epigenetic alteration influences gene expression, and thereby, gene function [6, 7].

DNA methylation has been studied extensively in relation to T2DM, and 3 systematic reviews have summarized the findings a few years back [8–10]. From systematic literature done till August 2015, Muka *et al.* [10] could not find any consistent association between global DNA methylation with T2DM, glucose, insulin and insulin resistance and reported epigenetic regulation of few candidate genes in blood cells, muscle, adipose tissue and placenta without any overlap between them. Walaszczyk *et al*. [9] could replicate association of methylation with T2DM in blood samples from the Lifelines study at 5 CpGs (in *ABCG1*, *LOXL2*, *TXNIP*, *SLC1A5* and *SREBF1*) out of the 52 CpGs they identified as reported to be differentially methylated in T2DM through a systematic review of the literature done till April 2017. Willmer *et al*. [8] also focused on differential methylation signatures in blood samples and reported *TCF7L2*, *KCNQ1*, *ABCG1*, *TXNIP*, *PHOSPHO1*, *SREBF1*, *SLC30A8* and *FTO* genes to be reproducibly associated with T2DM across multiple population groups in the literature reviewed between January 2002 and July 2018.

DNA methylation has been touted as a strong candidate biological process for identification of diagnostic and therapeutics for T2DM [11]. While the available systematic reviews have looked at DNA methylation associated with T2DM [8–10], they have not evaluated T2DM-associated DNA methylation comprehensively in all available human tissue and cell types. We set out to fill this research gap with the no time period cutoff until October 19, 2023, and including all available human tissue and cell types. We also report associated gene expression data, role of sex and ethnicity, in relation to DNA methylation in our review.

## Methods

### Searches

PubMed and Science Direct databases were independently searched by authors (NN, PN and JKV) using the key terms “type 2 diabetes mellitus” and “DNA methylation,” and their associated terms for all studies published up to October 19, 2023. All articles from the time of publication listing were considered, and as such no start date was set. No filters were applied during the search using the keywords, so as to not exclude any mislabeled/mis-annotated article type. The detailed search strategy is given in Additional file 1: Table S1.

### Study inclusion and exclusion criteria

The inclusion criteria were full-text English language articles on DNA methylation associated with T2DM in human subjects. Case–control and prospective studies investigating genome-wide methylation were included. Reviews, animal model studies, in vitro studies, irrelevant articles and articles published in other languages were excluded.

All participants, regardless of gender and ethnicity, classified as adults aged 18 years and above were included. All individuals who did not satisfy these criteria—children and adolescents under 18 years of age; as well as subjects with type 1 diabetes (T1DM) or gestational diabetes were excluded. As the association of DNA methylation with T2DM was the focus of this systematic review, intervention studies and clinical trials were excluded. Studies reporting association of DNA methylation with diabetes-related traits (hyperglycemia and insulin resistance) were retained.

All the articles were assessed for their eligibility based on their abstract or full text.

### Procedure

Disagreements between the authors, such as categorization and selection of articles, and data extraction, were resolved through discussion with AM. The Preferred Reporting Items for Systematic Reviews and Meta-Analyses (PRISMA) checklist was followed to represent the method used [12]. A total of 32 full-text articles are included in this systematic review.

The assessment of quality of the studies was done by adapting the New Castle Ottawa scale (NOS) [13]. The parameters used for the assessment are adequacy of case definition, representativeness of cases, selection of controls, definition of controls, comparability of cases and controls, ascertainment of exposure and method used for ascertainment of cases and controls. Scores were given to each of the included studies, and the total score was calculated according to the score sheet (NOS).

This review protocol was registered with the International Prospective Register of Systematic Reviews (PROSPERO) database (https://www.crd.york.ac.uk/prospero/) [14] (accessed April 18, 2023) (registration ID: CRD42020140436).

Pathologically connected pathways with differentially methylated genes in T2DM were analyzed using Kyoto Encyclopedia of Genes and Genomes (KEGG) and Jensen Disease database via Enrichr-KG [15].

## Results

**Fig. 1:**
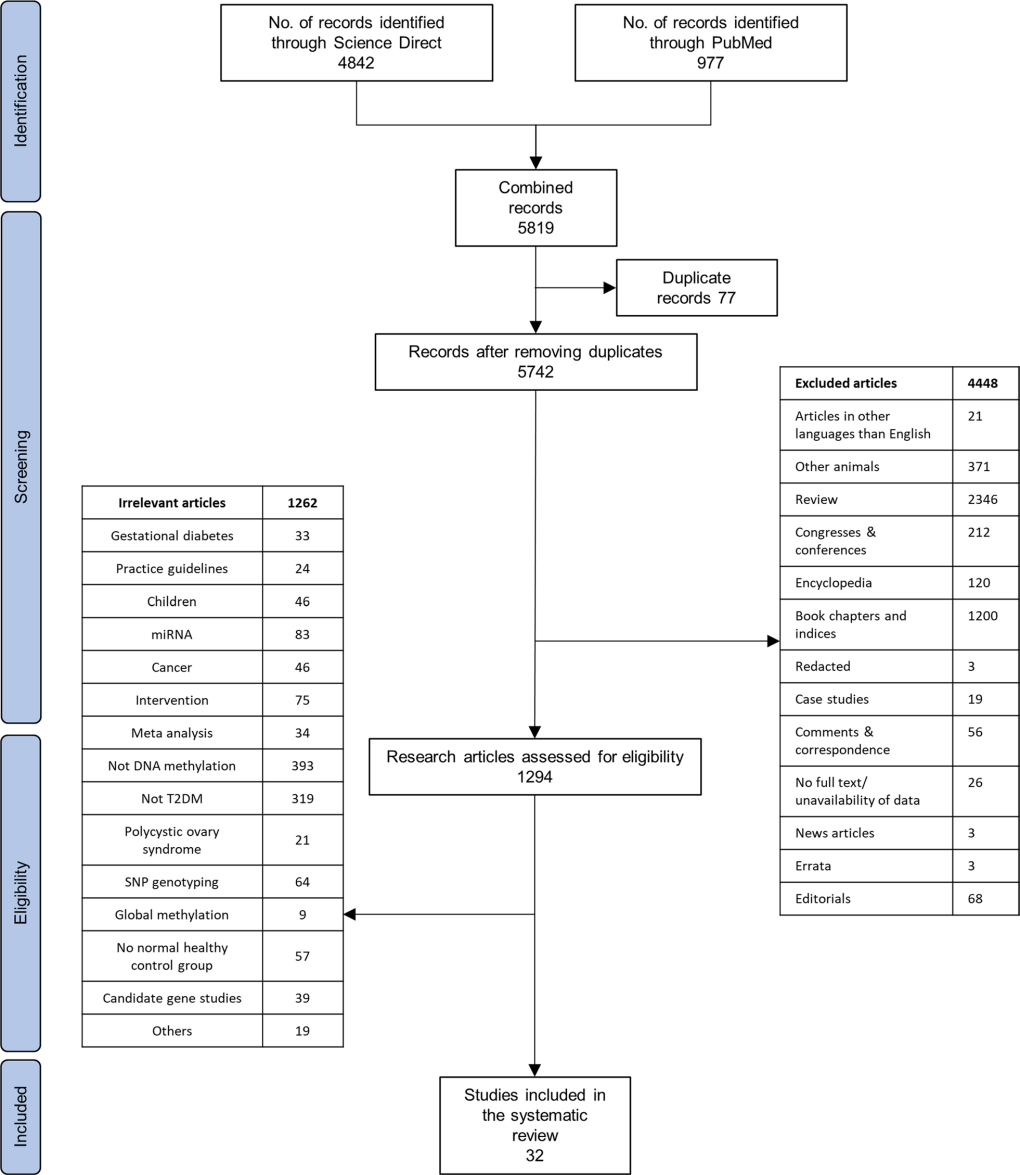
Preferred Reporting Items for Systematic Reviews and Meta-Analyses (PRISMA) [12] flowchart for the literature search process, performed up to October 19, 2023

We identified a total of 5819 articles during the initial search. Duplicates, irrelevant articles based on the study design, publication language, article type, and other articles not within our scope of review were removed. Thirty-two full-text articles were finally selected (Fig. 1).

NOS was used to access the quality of the articles. Of the 32 studies, 16 were assigned a score of more than 5, indicating high quality (Additional file 2: Table S2). As all the studies have used the same method of ascertainment for cases and controls, and the authors are not blinded to case–control status, these redundant scores are not presented. As the nonresponse rate was not available for any of the studies, this also has been omitted from the quality assessment table.

Case–control studies that reported differential DNA methylation between T2DM (case) and normoglycemic (control) subjects or reported associations between DNA methylation and clinical parameters related to glycemic control of the subjects (HbA1c, fasting blood glucose) and prospective nested case–control studies that reported differential DNA methylation measured at baseline/recruitment between subjects who developed T2DM (incident cases) and those that remained normoglycemic (control) during the follow-up period were finally included.

Participant details such as number of cases and controls and location of the study are also included. Details of the study participants who do not explicitly belong to either case or control group are also presented. The tissue source of the gene/loci identified in; method used for determining methylation status; and the validation method used for confirming the methylation status are tabulated in Table 1.

**Table 1:** Characteristics of studies included in this systematic review

Population			Study design	Location	Method used for DNA methylation measurement	Method used for validation	References
Control (M/F)	Type 2 Diabetes Mellitus (M/F)	Other (M/F)					
Blood							
7 pairs of healthy concordant twins	17 pairs of T2DM discordant twins; 3 pairs of T2DM concordant twins	–	Case–control, Twins study	UK	MEDIP	450 k	[53]
1119	329	–	Case–control	Germany	450 k	–	[18]
116/88	90/61	–	Case–control	Spain	450 k	EpiTYPER	[16]
215 Twins (discovery group) 250 Twins (replication group)	101 Twins (discovery group) 66 Twins (replication group)	–	Case–control, Twins study	China	450k	450 k	[17]
457	256	–	Case–control	Ghana	450 k	–	[20]
5/6	5/6	–	Case–control	UK	450 k	–	[101]
197/262	349/361	–	Prospective	Israel	Affymetrix SNP6 Microarray	Pyrosequencing	[58]
55/65	63/89	–	Case–control	China	MEDIP-Chip	–	[102]
98	94	–	Case–control	China	Affymetrix GeneChip Promoter 1.0R array	–	[103]
290	290	–	Prospective	Germany	EPIC BeadChip (850 k)	–	[70]
564	174	112 (Impaired glucose tolerance)	Case–control	USA	450 k	Pyrosequencing	[19]
5/5	15/15	–	Case–control	China	Affymetrix Axiom genome-wide TWB array	–	[56]
194/0	24/0	–	Case–control, Twins study	USA	450k	–	[21]
36/106	20/70	69/205 (Prediabetes)	Case–control	USA	EPIC BeadChip	–	[22]
350	385	–	Case–control	Taiwan	EPIC BeadChip	–	[23]
24/9	Short-term exposure T2DM: 25/9 Long-term exposure T2DM: 19/8	–	Case–control	France	EPIC BeadChip (850 k)	–	[24]
359/476 (Discovery cohort)	47/37 (Discovery cohort) (Controlled diabetics) 41/28 (Discovery cohort) (Poorly controlled diabetics)	–	Case–control	Germany	450 k	450k	[25]
172/268 (Replication cohort)	19/29 (Replication cohort) (Controlled diabetics) 13/26 (Replication cohort) (Poorly controlled diabetics)						
Adipose Tissue							
9/5 (Twins) 32/38 (Validation Cohort1) 15/13 (Cohort 2)	9/5 (Twins) 26/24 (Validation Cohort1) 15/13 (Cohort 2)	–	Case–control, Twins study	Sweden	450 k	450 k	[36]
Discovery cohort 0/10	Discovery cohort 0/8	–	Case–control	Spain	450 k	Bisulfite pyrosequencing	[37]
Validation cohort 14/41	Validation cohort 16/20						
12/0	12/0	–	Case–control	China	450 k	–	[38]
6/6	6/6	–	Case–control	Denmark	27 k	Bisulfite pyrosequencing	[39]
8/0	8/0	8/0 (Obese non-T2DM)	Case–control	Denmark	Reduced representation bisulfite sequencing	–	[40]
Pancreatic islets							
11/0	5/0	–	Case–control	Italy	27 k	Bisulfite pyrosequencing	[3]
Discovery cohort subgroup 1 (4/4)	Discovery cohort Subgroup 1 (4/4)	–	Prospective	Korea	450 k	Pyrosequencing	[27]
Discovery cohort Subgroup 2 (5/0)	Discovery cohort Subgroup 2 (5/0)						
Replication cohort (220)	Replication cohort (220)						
22/12	10/5	–	Case–control, Twins study	Sweden	450 k	Pyrosequencing	[28]
4/4	3/3	–	Case–control	–	450 k	Pyrosequencing	[59]
Liver							
60	35	–	Case–control	Finland	450 k	qPCR	[51]
0/96 (Discovery cohort)	0/96 (Discovery cohort)	–	Case–control	France	450 k	450 k	[50]
11/42 (Replication cohort)	11/42 (Replication cohort)						
Skeletal muscles							
17	17	8 (Impaired glucose tolerance)	Case–control	Sweden	MEDIP	Bisulfite sequencing	[52]
14	14	–	Case–control	–	450 k	–	[104]
9/0	13/9	2/7 (obese)	Case–control	USA	Reduced representation bisulfite sequencing	–	[105]
Spermatozoa							
9/0	8/0	–	Case–control	–	Next-generation sequencing (Illumina HiSeq 2000)	–	[57]

T2DM: Type 2 diabetes mellitus; 450 k: Illumina HumanMethylation450 BeadChip; 27 k: Illumina HumanMethylation27 BeadChip; 850 k: Illumina HumanMethylation EPIC BeadChip v1; qRT-PCR: Real-time quantitative reverse transcription PCR; MEDIP: Methylated DNA immunoprecipitation, Affymetrix Axiom genome-wide Taiwan BioBank (TWB) array

The loci/genes reported to be differentially methylated are tabulated in Table 2, where their methylation status is represented as downward arrow (hypomethylation) or upward arrow (hypermethylation). Wherever reported, the statistical significance of methylation (*P* value) is also mentioned. For studies reporting more than 10 differentially methylated genes, the top 5 hypo- and hypermethylated genes are listed.

**Table 2:** Differentially methylated genes/loci reported in T2DM subjects in case–control studies included in this review

Gene name	Methylation status in T2DM (compared to normoglycemic control subjects) ↑ ↓	*P* value			DNA methylation end point	Reference
		Univariate	After multiple testing correction			
Blood						
*MALT1*	↑		9.95 × 10^–10^ (discovery cohort)	Corrected for age, gender and genotype	Data normalized and linear regression done	[53]
	↑		2.0 × 10^–3^ (replication cohort)			
*GPR61*	↑		3.78 × 10^–6^ (discovery cohort)			
	↑		0.01 (replication cohort)			
*PRKCB*	↓		0.0384 (replication cohort)			
*PALLD* (cg03581271)	↑		0.047	Benjamin Hochberg values adjusted for age, sex, BMI, smoking, white blood cell proportion	Data normalized and linear mixed model used to assess DNA methylation and measures of glucose metabolism	[18]
*CREB3L2* (cg13016916)	↑		0.047			
*DGKZ* (cg17266233)	↓		0.047			
*EPB49* (cg03979241)	↑		9.4 × 10^–3^			
*ABCG1* (cg06500161)	↑		6.1 × 10^–3^			
*PXN* (cg11307565)	↑		0.021			
*KIAA0664* (cg11990813)	↑		0.034			
*TXNIP*	↓	1.17 × 10^−12^	5.0 × 10^−7^	Corrected for multiple testing	Average percent methylation	[16]
*TXNIP* (cg19693031)	↓		2.04 × 10^–9^ *	Adjusted for age, sex, BMI, smoking, alcohol consumption, BP, hypoglycemic drug use, surrogate variable	Methylation scores (β) ranged from 0 (unmethylated) to 1 (methylated)	[17]
*TXNIP*	↓	7.35 × 10^–18^	3.62 × 10^–12^	Corrected for multiple testing	Average percent methylation	[20]
*C7orf50*	↑	1.96 × 10^–09^	2.03 × 10^–03^			
*CPT1A*	↓	9.26 × 10^–08^	1.32 × 10^–02^			
*TPM4*	↓	3.44 × 10^–07^	3.69 × 10^–02^			
*CDC42EP2*	↑	6.36 × 10^–07^	4.64 × 10^–02^			
*VPS52*	↑	6.48 × 10^–07^	4.64 × 10^–02^			
*ELOVL5*	↓	0.0102 (Discovery stage)	-		Average percent methylation	[101]
	↓	0.0123 (Replication stage)				
*PRKCZ*	↑	< 0.01	-		Average percent methylation	[102]
*NR4A1*	↑	8.79 × 10^–06^	-		Model-based analysis of tiling arrays scores	[103]
*TXNIP*	↓		9.15 × 10^−25^	Bonferroni corrected values accounting 22 various tests	Methylation scores (β) ranged from 0 (unmethylated) to 1 (methylated)	[19]
*ABCG1*	↑		9.91 × 10^–11^			
*SAMD12*	↑		2.58 × 10^−8^			
*PTPRN2*	↑	8.79 × 10^–06^			Model-based analysis of tiling arrays (MAT) scores	[56]
*APBA1*	↑	8.79 × 10^–06^				
*LOC100288637*	↑	8.79 × 10^–06^				
*PIP5K1B*	↑	8.79 × 10^–06^				
*AFF2*	↑	8.79 × 10^–06^				
*SLIT2*	↓	8.79 × 10^–06^				
*MYO3B*	↓	8.79 × 10^–06^				
*PARP16*	↓	8.79 × 10^–06^				
*KIF18A*	↓	8.79 × 10^–06^				
*VPS13A*	↓	8.79 × 10^–06^				
*EFTUD2*	↓	8.79 × 10^–06^				
*TXNIP* (cg19693031)	↓		< 0.001	Adjusted for age, BMI, smoking status and peripheral blood leukocytes	Average percent methylation	[21]
*TXNIP* (cg19693031)	↓		4.43 × 10^−12^	Corrected for multiple testing	Average percent methylation	[22]
*OPTN* (cg02458882)	↑		3.78 × 10^−7^			
*cg21804949*	↓		5.28 × 10^−7^			
*CASKIN* (cg14955495)	↑		2.16 × 10^−6^			
*GPX6* (cg18890830)	↓		2.86 × 10^−6^			
*NELFCD* (cg22544867)	↓		4.35 × 10^−6^			
*ZNF350* (cg03577153)	↑		4.95 × 10^−6^			
*ATP10D* (cg14277924)	↓		8.16 × 10^−6^			
*ANKRD11* (cg02184744)	↓		3.35 × 10^−4^			
*FAM120AOS* (cg14471895)	↑		5.30 × 10^−4^			
*RASGEF1A* (cg06655623)	↑		8.09 × 10^−4^			
*TXNIP*	↓	< 0.001	*-*		Average percent methylation	[23]
*TXNIP* (cg19693031)	↓ (short-term T2D)	2.6 × 10^–4^			Methylation scores (β) ranged from 0 (unmethylated) to 1 (methylated)	[24]
	↓ (long-term T2D)	9.1 × 10^–5^				
*PTPRN2*	↑ (short-term T2D)					
*TXNIP (cg19693031)*	↓ (controlled diabetes)	–	0.046 (Discovery cohort) 0.001 (Replication cohort)	Adjusted for sex, BMI, age, smoking status, white blood cell composition and batch effect	Methylation scores (β) ranged from 0 (unmethylated) to 1 (methylated)	[25]
	↓ (poorly controlled diabetes)	–	1.7 × 10^−8^ (Discovery cohort) 0.0009 (Replication cohort)			
Adipose tissue						
*C1orf52* (cg21245975)	↓		0.006	Corrected for multiple testing	Average percent methylation	[36]
*MAD1L1* (cg23807071)	↓		0.006			
cg02166383	↓		0.000082			
*BCL2L14* (cg20141578)	↓		0.0004			
*MRGPRX2* (cg22051636)	↓		0.001			
*HLA-DPB1* (cg20223237)	↑		0.003			
cg16447950	↑		0.0007			
cg13117582	↑		0.0003			
cg26204682	↑		0.001			
*ARMS2* (cg25542438)	↑		0.004			
*HOOK2*	↑		1.1924 × 10^–07^	Corrected for multiple testing	Methylation scores (β) ranged from 0 (unmethylated) to 1 (methylated)	[37]
*ASTN2*	↑		6.8418 × 10^–08^			
*JMJD1C*	↑		7.8258 × 10^–06^			
*MIPEPP3*	↑		4.0028 × 10^–06^			
*PRSS50; PRSS45*	↑		9.4175 × 10^–06^			
*ACOT7*	↓		1.2085 × 10^–07^			
*PTPRN2*	↓		4.2091 × 10^–06^			
*SNAR-F*	↓		9.5658 × 10^–08^			
*SPON1*	↓		1.376 × 10^–08^			
*ZNF138*	↓		2.6298 × 10^–07^			
*MFSD1*	↑	< 0.05			Average percent methylation	[38]
*ARHGEF1*	↑	< 0.05				
*HNF4A* (cg19717150)	↑	0.0003	0.02	Adjusted for no. of probes tested		[39]
*CDKN2A* (cg12840719)	↑	0.003	0.02			
*L1TD1*	↓		1.11 × 10^–07^	Differentially methylated regions were found with a FDR cutoff of 10%	Average percent methylation	[40]
*BLOC1S4*	↓		2.53 × 10^–07^			
*LINC01558*	↓		6.29 × 10^–06^			
*FAM53A*	↓		1.14 × 10^–06^			
*PP14571*	↓		5.94 × 10^–07^			
*ANKS6*	↑		7.22 × 10^–09^			
*HDAC5*	↑		2.21 × 10^–08^			
*DNAAF5*	↑		5.53 × 10^–07^			
*KCNC3*	↑		8.36 × 10^–08^			
*MAFG*	↑		1.13 × 10^–07^			
Pancreatic islets						
*SFRS2IP*	↓	< 0.0001			Average percent methylation	[3]
*IIP45*	↓	< 0.0003				
*NTSR2*	↓	< 0.003				
*PCP4*	↓	< 0.003				
*CYP4F12*	↓	< 0.003				
*SCNN1D*	↓	< 0.003				
*CASP10*	↓	< 0.003				
*SLC7A11*	↓	< 0.003				
*PER2*	↑	< 0.003				
*VILL*	↑	< 0.03				
*B3GNT7*	↓	7.5 × 10^–6^			Average percent methylation	[28]
*BCOR*	↓	4.8 × 10^–5^				
*CDKN1A*	↓	1.2 × 10^–4^				
*FAM150B*	↓	2.7 × 10^–4^				
*TGFBR3*	↓	6.9 × 10^–5^				
*IL6ST*	↓	6.3 × 10^–5^				
*ZNF703*	↓	1.3 × 10^–4^				
*ANO8*	↑	3.0 × 10^–4^				
*DMTF1*	↑	1.6 × 10^–4^				
*SEMA5B*	↑	1.2 × 10^–4^				
DMR chr1:228,626,541:228,626,789	↑	–	–		Average percent methylation	[59]
chr11:115,500,818:115,500,941	↑	–				
chrX:25,022,180:25,022,280	↑	–				
chr12:188,286:188,865	↑	–				
chr10:58,384,121:58,384,364	↑					
chr6:105,401,793:105,401,826	↓	–				
chr15:32,319,709:32,319,757	↓	-				
chr11:82,403,487:82,404,321	↓	-				
chr18:112,930:113,037	↓					
chr9:65,522,265:65,522,281	↓					
Liver						
*ZNF23* (cg02772880)	↓	0.043	–		Average percent methylation	[51]
*RIPK4* (cg13520715)	↓	0.043	–			
*RIPK4* (cg01303480)	↓	0.048	**–**			
*ZNF295* (cg01303480)	↓	0.048	**–**			
*ZNF295* (cg13520715)	↓	0.018	–			
*CYB561D1* (cg19244300)	↓	0.033	–			
*IL23Ap19* (cg14940636)	↑	0.020	–			
*UPF2* (cg23421114)	↓	0.031	–			
*H19* (cg09575189)	↓	0.018	–			
*GOLPH4* (cg18142906)	↓	0.046	–			
*PDGFA*	↓	6.9 × 10^−7^ (Discovery cohort)	**–**		Average percent methylation	[50]
	↓	0.01 (Replication cohort)	**–**			
Skeletal muscles						
*PPARGC1A*	↑	< 0.05	**–**		Average percent methylation	[52]
*VPS39*	↓		< 0.05	FDR values adjusted for age, sex and BMI	Methylation scores (β) ranged from 0 (unmethylated) to 1 (methylated)	[104]
*TDP1*	↓					
*MAEA*	↓					
*FBN2*	↓					
*C21orf45*	↓					
*SND1*	↓					
*RNH1*	↓					
*ZNF415*	↓					
*AP2S1*	↓					
*WDR51A*	↓					
*EFHD1*	↑		0.00003	Adjusted for sex, BMI, age	Average percent methylation	[105]
*NIT2*	↑		0.00005			
*LONP1*	↑		0.00007			
*GLOD4*	↑		0.00014			
*NUBPL*	↑		0.00015			
*SLC25A37*	↓		0.00004			
*FASN*	↓		0.00005			
*PANK2*	↓		0.00006			
*USP30*	↓		0.00006			
*BOLA3*	↓		0.00016			
Spermatozoa						
*IRS* (chr2: 227,657,501–750)	↓	1.20 × 10^–11^	–		Average percent methylation	[57]
*PRKCE* (chr2: 46,108,751–900)	↑	7.21 × 10^–24^	–			
*PRKCE* (chr2: 46,156,251–500)	↓	1.32 × 10^–32^	–			
(chr16: 54,104,751–5000)	↑	2.04 × 10^–11^	–			
*PPARGC1A* (chr4: 24,024,251–500)	↓	9.67 × 10^–17^	–			
*PPARGC1A* (chr4: 24,111,501–750)	↑	3.14 × 10^–07^	–			
*KCNQ1* (chr11: 2,564,251–500)	↓	1.08 × 10^–10^	–			
*ATP10A* (chr15: 25,972,251–500)	↓	4.45 × 10^–73^	–			
*GHR* (chr5: 42,719,751–20,000)	↓	5.11 × 10^–13^	–			
*CREB1* (chr2: 208,466,751–7000)	↓	4.16 × 10^–13^	–			
*PRKAR1A* (chr17: 66,506,751–7000)	↓	8.03 × 10^–10^	–			
*HNF1B* (chr17: 36,106,501–750)	↑	1.73 × 10^–11^	–			

T2DM: Type 2 diabetes mellitus; ↑: Hypomethylation; ↓: Hypermethylation; FDR: False discovery rate; DMR: Differentially methylated region. *TXNIP cg19693031 reported to be negatively associated with fasting blood glucose

## Methods of DNA methylation analysis

Majority of the evaluated studies had employed array-based techniques to assess DNA methylation levels. Eighteen of 32 studies used Illumina 450 k array. Other array-based studies used Illumina 27 k array (2 studies), Illumina EPIC BeadChip array (4 studies; of which 2 studies specifically mentioned using the 850 k array—EPIC v1 array targeting 850 k probes), Affymetrix SNP6 microarray (1 study), Affymetrix GeneChip promoter 1.0R array (1 study) or Affymetrix axiom genome-wide Taiwan BioBank (TWB) array (1 studies). Rest of the studies used techniques such as methylated DNA immunoprecipitation (MEDIP) (2 studies), MEDIP-chromatin immune precipitation (1 study), reduced representation bisulfite sequencing (1 study) or next-generation sequencing (1 study) to measure DNA methylation levels.

### Tissues used in DNA methylation analyses

Of the 32 articles retrieved, 17 (53%) studies used blood samples, 4 (13%) studies used pancreatic islet samples, 5 (16%) studies used adipose tissue samples, 2 (6%) studies used liver samples, 1 (3%) study used spermatozoa samples and 3 (9%) used skeletal muscle samples for their DNA methylation analyses. None of the 32 studies reviewed here utilized more than one tissue from the same subjects for DNA methylation analyses.

### Genome-wide methylation analysis for T2DM

Of the 32 genome-wide methylation studies reviewed here, we identified a total of 130 loci that were differentially methylated between T2DM cases and controls across. In an instance where a study reports < 10 differentially methylated genes/loci, they are presented individually. However, in the case of a study which reports > 10 genes/loci, only the top 5 hypo- and 5 hypermethylated genes are highlighted for brevity and reported in Table 2. The direction of methylation (hyper- or hypomethylated in T2DM, compared to controls) and the reported *P* values (both unadjusted, and after multiple testing correction) have been included.

**Fig. 2:**
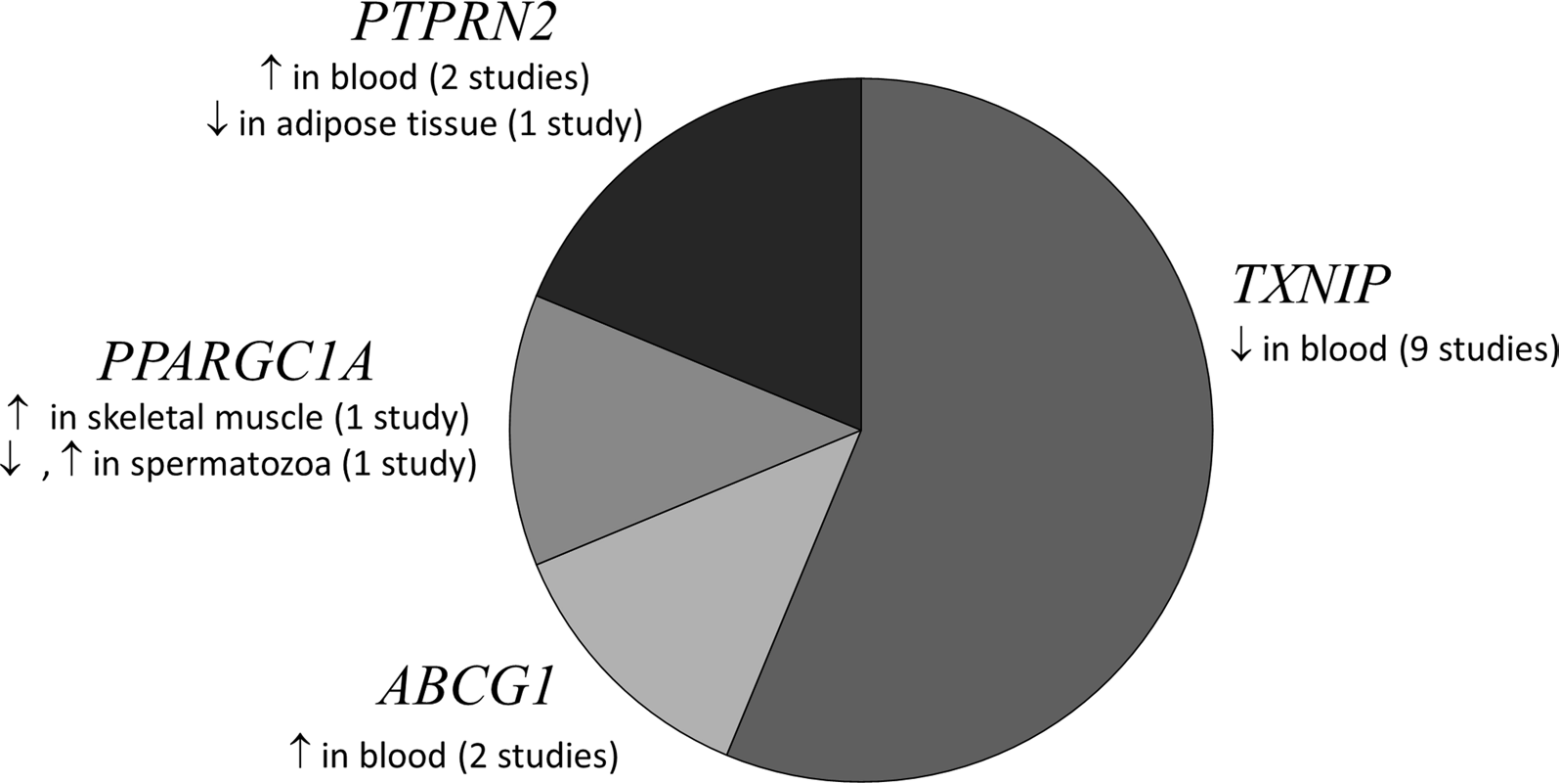
A pie chart depicting the genes that were consistently reported to be differentially methylated in ≥ 2 studies in various tissues from T2DM subjects. ↑: Hypomethylation, ↓: Hypermethylation in T2DM individuals compared to normoglycemics. *PPARGC1A* (chr4: 24,024,251–500) hypomethylated, (chr4: 24,111,501–750) hypermethylated in spermatozoa [57]

We identified genes such as *ABCG1, PPARGC1A*, *PTPRN2* and *TXNIP* with well-known T2DM genetic risk variants, which were consistently reported to be differentially methylated in more than one study (Fig. 2). Tissues used in identification of these gene were blood cells, liver, pancreatic islets and adipose tissue. *TXNIP* (cg19693031) was the most common gene identified consistently as hypomethylated in diabetic blood (9 studies). *TXNIP* also harbors established T2DM genetic risk variants [16, 17].

#### Blood

Although blood is not an insulin-responsive tissue, it is the prime minimally invasive tissue available for investigating T2DM-associated epigenetic markers. With the bulk (50%) of the studies coming from Europe, *ABCG1* [18, 19] and *TXNIP* [16, 17, 19–25] were some of the blood-based epigenetic markers which were reported to be associated with T2DM in more than one study. We were unable to find any study where differential methylation was investigated simultaneously in blood and other tissues from the same subjects.

#### Pancreatic islets

Insufficient secretion of insulin from pancreatic beta cells and increased secretion of glucagon from pancreatic alpha cells leads to development of T2DM and is known to be regulated by DNA methylation [26]. Three of the 32 studies, from Italy, South Korea and Sweden, included in this review have interrogated DNA methylation in pancreatic islets from T2DM individuals, donated after their death. Regions in *SFRS2IP* [3], *MSI2* [27], which are known to be associated with critical roles in nucleic acid binding, and *B3GNT7* [28] that is involved in synthesis of glycoprotein, were reported to be hypomethylated in pancreatic islets from T2DM individuals. Considering that DNA methylation can change based on the time of collection of tissue after death [29, 30], findings from these studies need to be interpreted in cognizance of the lack of details available in these studies about the cause of death or collection and storage of pancreatic islet tissue after death.

#### Adipose tissue

Adipose tissue is known to play a critical role in regulating body metabolism and energy homeostasis [31]. Dysregulation in adipose biology imposes serious health complications such as obesity and development of T2DM [31]. DNA methylation is an important regulator factor in development [32, 33] and dysfunction [34, 35] of adipose tissue. Five studies—4 of these representing the European population—included in this review have dissected whether T2DM, and related risk factors are associated with epigenetic modifications in human adipose tissue [36–40]. It is possible that DNA methylation alterations in these reported genes including *C1orf52* [36], *HOOK2* [37], *MFSD1* [38], *HNF4A* [39] and *L1TD1* [40] contribute to or are caused by T2DM.

*C1orf52* is involved in RNA binding in adipose tissue [41], and *HOOK2* is responsible for cytoskeleton maintenance via regulation of microtubules [42], while *MSFD1* regulates lysosome transport [43]. Epigenetic alterations in such genes involved in cell structure and function can cause dysfunction in adipose tissue, thereby leading to insulin resistance. While *HNF4A* mainly regulates transcription in hepatocytes and is associated with Fanconi renotubular syndrome 4 with maturity-onset diabetes of the young [44] and maturity-onset diabetes of the young, type 1 [45], it is also known to play a role in lipid and glucose metabolism [46, 47]. *L1TD1* is predicted to be involved in single-stranded RNA-binding activity [48].

#### Liver

Liver is known to be involved in regulating glucose level by storing and releasing glycogen in response to insulin and glucagon [49]. Impaired hepatic gluconeogenesis, glycogenolysis and insulin sensitivity are known to play an important role in T2DM development and other risk factors. Altered hepatic metabolism could be the cause or consequence of DNA methylation modification. Genes involved in intracellular tyrosine kinase activity—*PDGFA* [50], transferring phosphorus-containing groups and protein tyrosine kinase activity—*RIPK4* [51], heme binding and oxidoreductase activity—*CYB561D1* [51], were found to be hypomethylated in the diabetic groups. However, the gene involved in inflammation—*IL23Ap19* [51] was identified to be hypermethylated in the diabetic group. Of the two studies reported here, one was from France and the other from Finland.

## Gene expression studies

Out of the 32 studies reviewed, 8 had also examined differences in gene expression between T2DM and normoglycemic individuals. To examine if increase in methylation of a gene causes decrease in expression of that gene, we analyzed the studies that report both differentially methylated genes and gene expression, in the same population and study setting, using tissues from the same study participants (Table 3). For most of the loci with both DNA methylation and gene expression data available, we found that increase in methylation was associated with decrease in expression, concurrent to the current understanding [6]. Hypermethylation of *PPARGC1A* in skeletal muscles [52], *ABCG1* in blood [18] and *PER2* in pancreatic islets [3] was associated with lower expression of the corresponding genes.

**Table 3:** Differentially methylated genes/loci and associated gene expression levels in T2DM subjects, from case–control studies included in the review

Gene name	Methylation status ↑ ↓	Gene expression	Gene expression P value	Method	References
Blood					
*ABCG1*	↑	↓	1.5 × 10^–9^	Illumina Human HT-12 v3 Expression BeadChip	[18]
*PRKCZ*	↑	↓	< 0.05	Western Blotting (protein level)	[102]
*NR4A1*	↑	↓	< 0.05	qRT-PCR	[103]
*NT5C2*	↑	↓	0.05	qRT-PCR	[56]
Adipose tissue					
*S100A4*	↓	↑	0.005	qPCR	[36]
*SLC37A2*	↑	↓	0.005		
Pancreatic islets					
*PER2*	↑	↓	< 0.05	GeneChip Expression microarray	[3]
*SFRS2IP*	↓	↑	< 0.05		
*PTPRD*	↓	↑	< 0.05		
*HAPLN1*	↓	↑	< 0.05		
*FLJ14054*	↓	↑	< 0.05		
*SCNN1D*	↓	↑	< 0.05		
Liver					
*PDGFA*	↓	↑	< 0.007	qRT-PCR	[50]
Skeletal muscle					
*PPARGC1A*	↑	↓	< 0.036	qPCR	[52]

In the DNA methylation column ↑: hypermethylation; ↓: hypomethylation; in the gene expression column ↑: increased expression; ↓: decreased expression; qPCR: real-time polymerase chain reaction; qRT-PCR: real-time-reverse transcription polymerase chain reaction

### Twin studies

Five of the 32 studies reviewed here have investigated DNA methylation in monozygotic twin cohorts [17, 21, 28, 36, 53] (Table 4). *MALTI* [53] which is known to be involved in energy and insulin signaling pathways [54], *PTBP1* [36] that is involved in nucleic acid binding, and *ANO8* [28] that is involved in calcium transport, were hypermethylated in diabetic twins in peripheral blood, adipose tissue and pancreatic islets, respectively. *TXNIP* [17, 21], *COL21A1* [36] and *B3GNT7* [28] were hypomethylated in blood cells, adipose tissue and pancreatic islets, respectively, from the diabetic twins. Dayeh *et al*. reported differential methylation of *ABCG1* (hypermethylated in blood and adipose tissue) and *PHOSPHO1* (hypomethylated in skeletal muscle) in monozygotic twins discordant for T2DM [55].

**Table 4:** Differentially methylated genes/loci reported in T2DM subjects in studies with twins as participants

Gene name	Methylation status ↑ ↓	*P* value	Population			References
			Control M/F	T2DM M/F	Others M/F	
*COL21A1*	↓	0.001	9/5 (twins) Validation Cohort1 32/38 Cohort 2 15/13	9/5 (twins) Validation Cohort1 26/24 Cohort 2 15/13	–	[36]
*STK24*	↓	0.003				
*CUX1*	↓	0.01				
*TANK*	↓	0.01				
*CFDP1*	↓	0.01				
*PTBP1*	↑	0.01				
*GSTM5*	↑	0.01				
*MGRN1*	↑	0.001				
*RNF170*	↑	0.04				
*CREBBP*	↑	0.02				
*MALT1*	↑	9.95 × 10^−10^	7 pairs of healthy concordance twins	17 pairs of T2DM discordant twins and 3 pairs of T2DM concordant twins	–	[53]
*GPR61*	↑	0.012				
*PRKCB*	↑	0.038				
*B3GNT7*	↓	7.5 × 10^–6^	22/12	10/5	–	[28]
*BCOR*	↓	4.8 × 10^–5^				
*CDKN1A*	↓	1.2 × 10^–4^				
*FAM150B*	↓	2.7 × 10^–4^				
*TGFBR3*	↓	6.9 × 10^–5^				
*IL6ST*	↓	6.3 × 10^–5^				
*ZNF703*	↓	1.3 × 10^–4^				
*ANO8*	↑	3.0 × 10^–4^				
*DMTF1*	↑	1.6 × 10^–4^				
*SEMA5B*	↑	1.2 × 10^–4^				
*TXNIP*	↓	< *0.001*	194/0	24/0	–	[21]
*TXNIP*	↓	*2.04* × *10*^–9^*	215 twins (discovery group) 250 twins (replication group)	101 twins (discovery group) 66 twins (replication group)	–	[17]

In the DNA methylation status column ↑: hypermethylation; ↓: hypomethylation. *TXNIP associated with fasting blood glucose

### Association between diabetes related traits and DNA methylation

Only 4 of the 32 studies reported association between diabetes-related traits (hyperglycemia and insulin resistance) and DNA methylation [17–19, 22]. Kriebel *et al*. reported significant association between measures of glucose metabolism phenotypic traits and methylation levels of 31 CpG sites in PBMCs [18]. Five CpGs were found to be associated with fasting glucose, 1 CpG with 2-h glucose, 8 with fasting insulin and 26 with Homeostatic Model Assessment of Insulin Resistance (HOMA-IR) in model 1 (Table 2) [18]. There was no significant association between HbA1c and DNA methylation levels in model 1; in model 2, after adjustment for body mass index (BMI), the effect strength was reduced by 30% for DNA methylation associations with fasting glucose suggesting that the associations between DNA methylation and diabetes-related traits are partially mediated by BMI [18].

Kulkarni *et al*. investigated association between 446,356 autosomal CpG sites and phenotypic traits in PBMCs, of which a total of 51 CpG sites were significantly associated with T2DM, 19 with FBG and 24 with HOMA-IR (Table 2) [19].

Wang *et al*. report association between 63 differential methylated loci and fasting blood glucose and association between 6 differentially methylated loci with HbA1c in blood samples from twins discordant for diabetes [17]. Among these, hypomethylation of *TXNIP* [17, 19] and hypermethylation of *ABCG1* [18, 19] were positively associated with fasting blood glucose (FBG), and hypermethylation of *SAMD12* was negatively associated with FBG [19]. *TXNIP* hypomethylation in blood cells was found to be associated with hyperglycemia in individuals from Taiwan [23], France [24], the USA [21] and China [17].

Dawes *et al*. performed genome-wide DNA methylation on blood samples from normoglycemic (n = 142), pre-diabetic (n = 274) and diabetic (n = 90) individuals [22]. They identified HbA1c-associated DNA methylation loci by regressing the probes against HbA1c values, while controlling for age, sex and BMI [22]. They report cg19693031 (*TXNIP*) as the locus most highly associated with HbA1c [22].

### Enrichment analysis of genes differentially methylated in T2DM

**Fig. 3:**
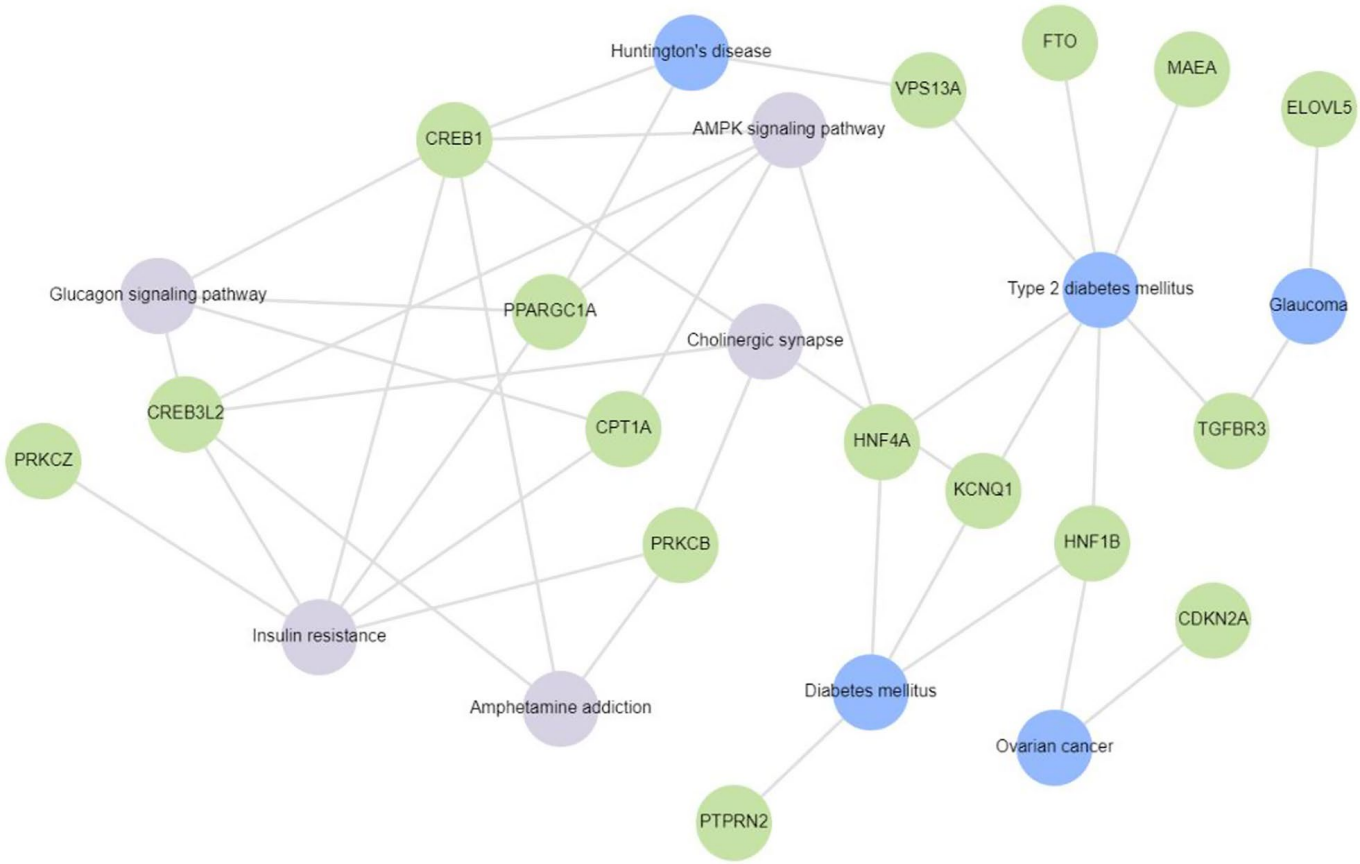
Gene enrichment analysis of 17 of the 130 genes reported to be differentially methylated in T2DM subjects in the 32 studies included for review using Enrichr-KG. These genes were mapped to diabetes and related disorders. Insulin resistance, glucagon signaling pathway, glaucoma, AMPK signaling pathway, cholinergic synapse, ovarian cancer, amphetamine addiction and Huntington’s disease were found to be associated with *KCNQ1*, *FTO*, *PPARGC1A*, *PTPRN2*, *ELOVL5*, *HNF1B*, *HNF4A*, *VPS13A*, *MAEA*, *CREB1*, *CPT1A*, *PRKCZ*, *PRKCB*, *CREB3L2*, *CDKN2A* and *TGFBR3*

**Fig. 4:**
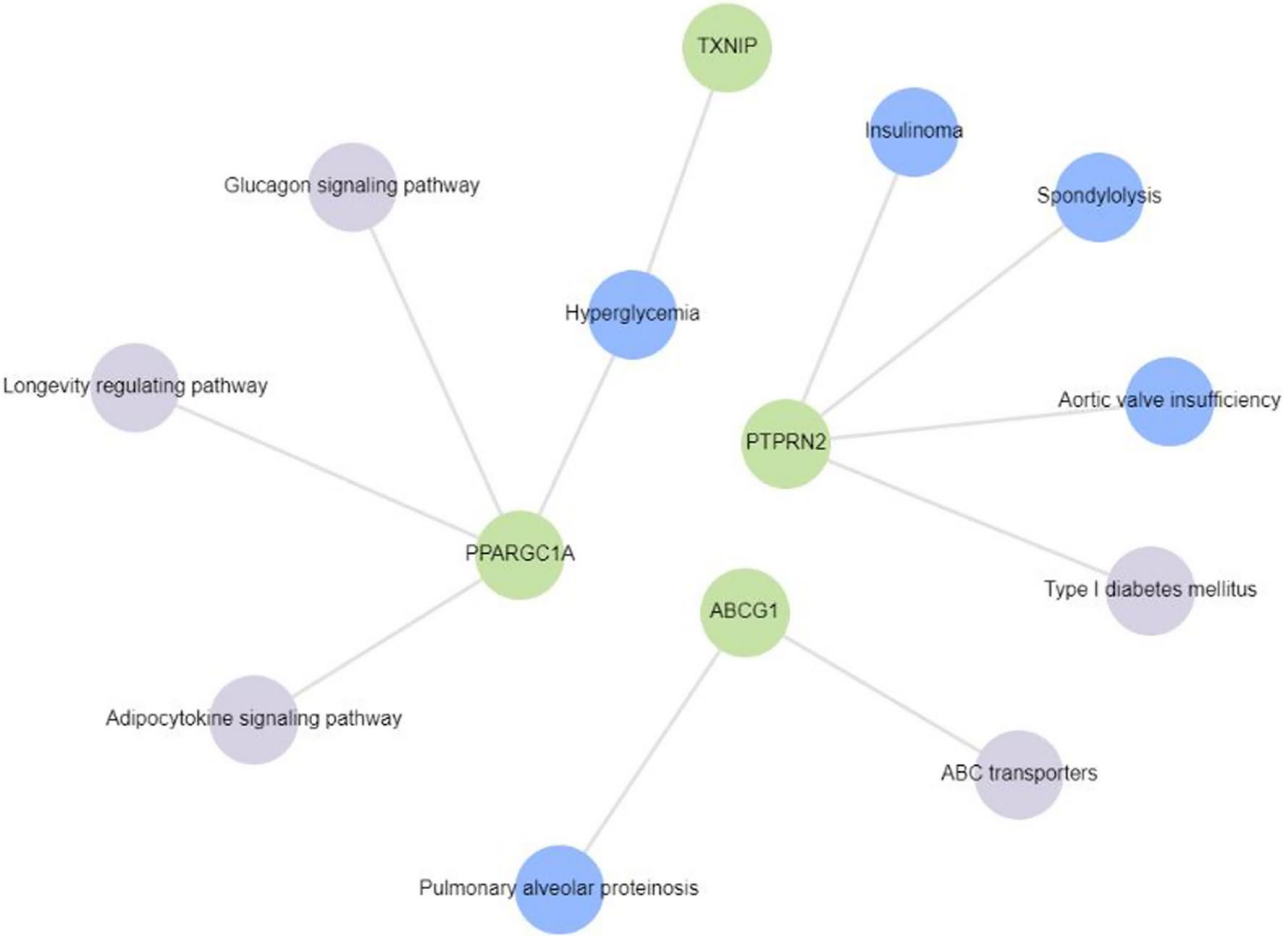
Gene enrichment analysis of 4 genes reported to be differentially methylated in T2DM subjects in > 1 study from among the 32 studies included for review using Enrichr-KG. Hyperglycemia, type 1 diabetes, adipocytokine signaling pathway, glucagon signaling pathway, longevity regulating pathway and ABC transporters were found to be associated with *PPARGC1A*, *TXNIP*, *PTPRN2* and *ABCG1*

Enrichment analysis of signaling pathways relevant to the pathophysiology of T2DM using Enrichr-KG [15] was done in two steps. Initially, all 130 genes differentially methylated in T2DM in all 32 studies reviewed were included (Fig. 3). To take into account reproducibility of these findings, enrichment analysis was separately done specifically for the genes (*ABCG1*, *TXNIP*, *PTPRN2*, *PPARGC1A*) that were reported to be differentially methylated in T2DM in more than one study (Fig. 4). *TXNIP* hypomethylation in blood was linked to hyperglycemia. *PPARGC1A* hypermethylation in skeletal muscles, and two CpG sites that were hyper- and hypomethylated, respectively, in spermatozoa, was linked to hyperglycemia and adipocytokine signaling pathway. *PTPRN2* that was reported to be hypermethylated in blood and hypomethylated in adipose tissue was associated with T2DM and T1DM.

### Subgroup analysis based on ethnicity

Out of the 32 studies, 16 (50%) were from Europe, 4 (13%) were from North America, 8 (25%) were from Asia and 1 (3%) from Africa. Three studies (9%) did not report their subjects’ ethnicity/demography.

*TXNIP* was the most commonly reported hypomethylated gene in blood cells of T2DM individuals from all the geographic locations [16, 17, 19–24]. *ABCG1* was found be to hypermethylated in blood cells of type 2 diabetics in studies from Europe [18] and the USA [19]. *PTPRN2* was reported to be hypermethylated in peripheral blood in studies from China [56] and France [24]. Conversely, *PTPRN2* was reported to be hypomethylated in adipose tissue from a Spanish study [37].

### Subgroup analysis based on sex

*PPARGC1A* was assessed for differential methylation in two studies which had only male participants [52, 57]. *PPARGC1A* was hypermethylated in skeletal muscle of T2DM men [52]. Of the two differentially methylated regions in *PPARGC1A* identified in sperm, chr4: 24,111,501–750 was reported to be hypermethylated, and chr4: 24,024,251–500 was reported to be hypomethylated [57]. We did not find other epigenome-wide studies that report differential methylation of *PPARGC1A* in female-only or mixed-sex populations.

*PDGFA* was found to be hypomethylated in hepatocytes from liver biopsies of female T2DM participants of the discovery group and was later confirmed in both men and women by Abderrahmani *et al*. [50]. Similarly, hypomethylation of *MSI2* in blood cells was first observed in a discovery group comprised of only men, and then in a replication group of both men and women by Jeon *et al*. [27].

In the cg 11,738,485-region (5 CpG nucleotides) of *HOOK2*, female T2DM visceral adipose tissue samples were hypermethylated, while male T2DM samples were hypomethylated, compared to non-diabetic sex-matched control samples [37]. None of the other loci/genes were reported to be differentially methylated in a sex-specific manner.

### Internal and/or external validation

Only 22% of the studies reviewed (7 out of 32) validated their findings in an independent set of subjects using the same DNA methylation measurement method that they had used for the discovery set of samples [17, 25, 27, 36, 37, 50, 53]. Others used either bisulfite pyrosequencing/sequencing (10 studies) [3, 19, 27, 28, 37, 39, 52, 58–60], qPCR (1 study) [51], EpiTYPER (1 study) [16], Illumina 450 k (3 studies) [36, 50, 53] or MEDIP (1 study) [61] for their internal validation. Sixteen studies (50%) did not perform any validation for their findings.

### Replication for case–control studies

We later looked for candidate-gene DNA methylation studies to see if the differentially methylated genes found in genome-wide studies have been confirmed in them. The following genes were reported to be differentially methylated in T2DM compared to normoglycemic controls in independent candidate-gene DNA methylation studies in the same tissue as the initial discovery group—*ABCG1 *[62, 63], *FTO* [64–66], *TXNIP* [67] and *KCNQ1* [64, 68] in PBMCs, and *PPARGC1A* in pancreatic islets [69].

### Prospective studies

As prospective studies observe the disease condition over a long period, they help in better understanding the role of a gene/set of genes toward pathogenesis. In our review, we came across three such studies that looked at incidence of T2DM and epigenetic modifications in genes associated with this incidence (Table 5).

**Table 5:** Genes differentially methylated at baseline/recruitment in normoglycemic subjects who developed T2DM during follow-up in prospective studies

Gene name	Methylation status in T2DM (compared to normoglycemic control subjects) ↑ ↓	*P* value			DNA methylation end point	References
		Univariate	After multiple testing correction			
Blood						
*IGFBP2* (cg005689321)	↑	–	< 0.05	FDR corrected		[70]
*IGFBP2* (cg03625261)	↑	–	< 0.05			
*IGFBP2* (cg26187237)	↑	–	< 0.05			
*IGFBP2* (cg25316969)	↑	–	< 0.05			
*IGFBP2* (cg25380868)	↓	–	< 0.05			
*IGFBP2* (cg13220299)	↓	–	< 0.05			
*IGFBP2* (cg03149532)	↓	–	< 0.05			
*THADA* (chr2: 43,590,864)	↓	0.012	0.0464	Corrected for multiple testing	Average percent methylation	[58]
*JAZF1* (chr7: 28,143,482)	↓	0.0034	0.0188			
*SLC30A8* (chr8: 118,257,326)	↓	0.0025	0.0188			
*SLC30A8* (chr8: 118,257,358)	↓	0.0102	0.0428			
*SLC30A8* (chr8: 118,258,573)	↓	0.0027	0.0188			
*TCF7L2* (chr10: 114,734,658)	↓	0.0012	0.0116			
*TCF7L2* (chr10: 114,739,401)	↓	0.0079	0.0361			
*TCF7L2* (chr10: 114,743,601)	↓	0.0004	0.0055			
*TCF7L2* (chr10: 114,743,664)	↓	0.0001	0.0025			
*KCNQ1* (chr11: 2,805,916)	↓	0.0033	0.0188			
*KCNQ1* (chr11: 2,806,049)	↓	0.0001	0.0015			
*KCNQ1* (chr11: 2,806,079)	↓	0.0038	0.0192			
*FTO* (chr16: 52,366,732)	↓	1 × 10^–5^	0.0006			
Pancreatic islets						
*MSI2*	↓	0.013		Methylation scores (β) ranged from 0 (unmethylated) to 1 (methylated)		[27]

↑: Hypomethylation; ↓: Hypermethylation; FDR: False discovery rate

In a 1:1 matched nested case–control study of 290 incident diabetics, who developed T2DM and 290 controls, who remained normoglycemic during the 4-year follow-up, baseline methylation at 7 CpG sites of *IGFBP2* in blood cells (4 hypermethylated and 3 hypomethylated in cases) was associated with increased risk of incident T2DM [70].

Jeon *et al*. reported that differential methylation of three CpG sites in blood cells at baseline was associated with T2DM/hyperglycemia after a 10-year follow-up [27]. These CpG sites were cg23586172 (annotated to *MSI2*, hypomethylated), cg22604213 (annotated to *CXXC4,* hypomethylated) and cg25290098 (hypomethylated) in T2DM [27]. They further reported *MSI2* hypomethylation in a replication group of 220 normoglycemic and 220 T2DM individuals [27]. Furthermore, whole-genome bisulfite sequencing of pancreatic islets of 2 T2DM and 16 normoglycemic individuals revealed that chr17:55,484,635 in *MSI2* was hypomethylated in T2DM [27]. While *MSI2* hypomethylation was seen in both pancreatic islets and PBMCs, pancreatic islets showed increased difference of 16% methylation versus 3% in PBMCs of *MSI2* in T2DM when compared to normoglycemics [27]. *MSI2* differential methylation was not found to be replicated in locus-specific case–control studies.

From the Jerusalem LRC longitudinal study, Toperoff *et al*. selected 58 individuals who developed impaired glucose metabolism over a 13-year follow-up and reported hypomethylation of a single CpG site in the first intron of *FTO* in peripheral blood samples collected at baseline [58]. Chen *et al*. similarly reported hypomethylation of *FTO* in their case–control study [57].

In a longitudinal study of Indian Asians living in London, UK (1074 incident T2DM and 1590 normoglycemic controls), over 8 years of follow-up, Chambers *et al*. reported that DNA methylation levels of *TXNIP*, *PROC*, *C7orf29*, *SREBF1*, *PHOSPHO1*, *SOCS3* and *ABCG1* in blood cells were positively associated with future T2DM incidence [71]. Of these, higher baseline methylation levels in *TXNIP*, *SREBF1*, *PHOSPHO1*, *SOCS3* and *ABCG1* were also associated with incident T2DM in an European cohort of 377 incident T2DM and 764 normoglycemic individuals [71].

#### Differential methylation in animal models

To check if animal model studies exist that have investigated or reported differential methylation in the genes identified as differentially methylated in the human case–control studies as playing causal or mechanistic role in the development of T2DM, a simple literature search was done using PubMed and bibliography search. A study in rat pancreatic islets reported Kcnq1 was hypomethylated in older rats (15 months of age) when compared to younger rats (3 months of age), but this difference was not statistically significant, while there was no comparison done with a rat T2DM model [72]. Though Toperoff *et al*. reported hypomethylation of *KCNQ1* in blood cells [58], there are no human pancreatic islet studies reporting hypomethylation of *KCNQ1*. Identification of multiple variants in genome-wide association studies [73–81] points toward the likely importance of *KCNQ1* in T2DM pathophysiology.

High-fat diet was shown to induce hypermethylation of Tcf7l2, and subsequently, gene expression was decreased in mouse islets [82]. This is in contrast to the findings where *TCF7L2* is hypomethylated in T2DM human blood cells [58] and pancreatic islets [59]. It is to be noted that the mice used were non-diabetic adult males aged 8 weeks (equivalent to middle-aged humans [83]) [82], while the human study group were a mix of men and women aged about 58–65 years, and for the human pancreatic islet study, the samples had been collected post-mortem [58, 59]. Although there is an inverse differential methylation status among mice and humans, it is important to note that a high-fat diet caused suppression of Tcf7l2 gene expression and thus decreases pancreatic beta-cell survival (mediated via the transcription of Wnt/Beta-catenin signaling pathway [84]) [82].

## Discussion

From the 32 studies finally included for this systematic review, we identified 130 genes with T2DM-associated differential methylation across all tissues analyzed. These comprise of the top 5 hypo- and hypermethylated genes for studies reporting more than 10 differentially methylated genes/loci. Of these 130 genes, 4 (3%; *ABCG1, PPARGC1A*, *PTPRN2* and *TXNIP*) were reported in > 1 studies. The genes and associated pathways with altered DNA methylation in T2DM are conceptually summarized in Fig. 3 (for 16 of the 130 genes, for which pathway analysis could be conducted) and Fig. 4 (for the 4 genes reported to be differentially methylated in > 1 studies).

Previous systematic reviews [8, 9] have reported differentially methylated loci in genes in T2DM blood cells including *ABCG1*, *TXNIP*, *KCNQ1*. While another such review by Muka *et al*. reported several epigenetically regulated genes from blood cells, adipose tissue, muscle and placenta, there was no overlap between them, and no association was found between global DNA methylation and T2DM/hyperglycemic markers [10].

We did not limit our search to a particular method used to identify DNA methylation, and several studies included have used Illumina’s 450 k array. The common method of validation/replication in the studies reviewed here was bisulfite pyrosequencing. We also looked at candidate-gene DNA methylation studies which aimed to replicate/validate the epigenome-wide studies reviewed here and found that in blood cells, *ABCG1* [62], *FTO* [64] and *KCNQ1* [64] were hypermethylated, while *TXNIP* was hypomethylated [67]. *TXNIP* codes for thioredoxin-interacting protein, and this protein plays a major role in pathways generating reactive oxygen species [85], regulating redox-dependent signaling pathways, mediating oxidative stress, suppressing cell growth and inducing pancreatic beta-cell apoptosis [86]. *ABCG1* codes for the protein responsible for intracellular sterol transport [87], and it regulates cholesterol efflux from macrophages to high-density lipoprotein in diabetics [88], indicated by altered lipid levels [89]. While genetic variants and epigenetic modification of *KCNQ1* have been linked with T2DM via whole body insulin sensitivity [90], there is no clear evidence for the mechanistic link. Likewise, there has been no clear evidence of *FTO* link with T2DM.

As gene expression is known to be regulated by DNA methylation, it is important to validate this claim in the epigenome-wide association studies. We were able to report the relation between DNA methylation in the promoter region and expression of the corresponding gene, as none of the studies had mentioned methylation status of other regions of the genes. Of the studies reviewed here, we found that DNA methylation of genes was inversely related to gene expression. For example, hypomethylation of *S100A4* in adipose tissue [36] and *PDGFA* in hepatocytes [50] was associated with increased expression of these genes, and hypermethylation of *PPARGC1A* in skeletal muscles [52], *ABCG1* in blood [18] and *PER2* in pancreatic islets [3] was associated with lower expression of the corresponding genes. Even though we observed DNA methylation being related inversely with expression of the corresponding gene in the studies reviewed, this is not a rule as has been reported repeatedly [91]. It is also important to note that there have been reports of methylation levels differing between different regions of the gene that influence gene expression; for instance, Anastasiadi *et al*. recently reported that gene expression is dependent on methylation of the first exon, more than methylation of the promoter region [92]. Moreover, in other studies such as one by Ball and colleagues, highly expressed genes have been reported to have low methylation levels in the promoter region and high methylation levels in rest of the gene body [93]. We could not, however, evaluate the relations between DNA methylation in various regions of a gene and its corresponding expression in this study since the studies reviewed by us have reported DNA methylation specifically in the promoter region.

Epigenetic studies on twins discordant for disease status are crucial in understanding the genetic basis of epigenetic differences observed in cross-sectional studies. Of the 5 studies included in our search, 3 did not have any common differentially methylated genes among them, while the other two studies that used blood cells as the source tissue had *TXNIP* as the common differentially methylated gene between them, with hypomethylation of *TXNIP* in diabetic blood samples observed in both these studies [17, 21]. *TXNIP* is the only gene reported to be hypomethylated in diabetic blood in both case–control studies [55] and in twin studies [17, 21] where the influence of underlying genetic factors is not masked. *TXNIP* has also been reported to be hypomethylated in diabetic pancreatic islets [55] and skeletal muscle [55], making it a potentially important causal gene in the pathophysiology of T2DM.

T2DM is known to be associated with other comorbidities such as obesity and cardiovascular complication. These comorbidities share some common risk factors like age, BMI and cholesterol content in blood. These risk factors are influenced by genes such as *KCNQ1*, *TCF7L2* and *FTO* [94]. Other systematic reviews have looked at epigenetic changes in obesity [95], aging [96, 97] and cardiovascular complications [98]. Andrade *et al*. aimed to identify epigenetic changes in human adipose tissue from obese/overweight individuals with and without metabolic disorders like T2DM [95]. They also report differentially methylated genes that we have been reported in this review, such as *KCNQ1*, *FASN*, *MFSD1*, *TXNIP*, *PPARG*, *IRS1* and *TCF7L2*, from the same studies [95]. Krolevets *et al*. report that in addition to about 75,000 CpG sites and 19,000 genes, *PTPRN2* was among the most frequently reported gene that was associated with cardiac disorders, although the direction of methylation is not specified [98]. Of the two studies that investigated DNA methylation in aging [96, 97], no genes/CpG sites/studies were common with the ones mentioned in our review.

One of the most important factors in looking at T2DM as an epidemic is the geographic location of the site of reported data. With a large amount of data coming in from Europe alone, it is important to perform similar studies in other parts of the world and including various other ethnic groups to validate these reports and also help in mapping the genetic diversity to be able to tackle T2DM. India being the most populous country [99] with about 11% of Indians suffering from T2DM (in 2020) [100], it is imperative to study this population to uncover T2DM susceptible loci/genes. Of note, Chambers *et al*. have followed up London resident Indian Asians, for 8 years, and found that DNA methylation levels of *TXNIP*, *PROC*, *C7orf29*, *SREBF1*, *PHOSPHO1*, *SOCS3* and *ABCG1* were positively associated with future T2DM incidence [71], but similar studies are lacking in Indians living in India, where exposure to pollution and availability and consumption of healthy diet are vastly different.

As for sex-specific methylation signatures of T2DM, differences were not seen between men and women except in genes *HOOK2* [37] and *MSI2* [27], which were hypermethylated in adipose tissue, and hypomethylated in blood, respectively*.* Finally, we searched if the genes which we found to be highly reported to be differentially methylated in human were also reported to be differentially methylated in animal models. *KCNQ1* was reported to be hypomethylated in both T2DM human [58], and older mice model compared with younger mice [72] suggesting age-related methylation changes across species. In both humans [58], and mice fed with a high-fat diet, *TCF7L2* was hypomethylated, and this DNA methylation change in mice was induced because of their diet [82], suggesting that nutrient consumption plays a role in epigenetic modification of genes involved in beta-cell function, and a healthy diet can have a protective role in maintaining homeostasis.

Although we did not look at clinical trials and candidate-gene studies that report differential DNA methylation, our review is an up-to-date report of epigenome-wide studies that includes prospective studies. We also report gene expression data in comparison with DNA methylation. Furthermore, a systematic report of differentially methylated gene/loci in tissues including blood cells, adipose tissue, pancreatic islet, skeletal muscles, liver and spermatozoa is included. While sex and ethnicity play a major role in pathology, we have tried to highlight these effects.

As with previous reviews, we emphasize the need for more prospective studies and replication of genome-wide association studies in different tissue types and populations.

## Conclusion

From the 32 studies that report differentially methylated genes/loci between T2DM and normoglycemic individuals, *ABCG1* (hypermethylated in blood), *FTO* (hypermethylated in blood and spermatozoa), *KCNQ1* (hypermethylated in blood and hypomethylated in spermatozoa), *TXNIP* (hypomethylated in blood), *PPARGC1A* loci at chr4: 24,111,501–750 (hypermethylated in skeletal muscle and spermatozoa) and loci at chr4: 24,024,251–500 (hypomethylated in spermatozoa), *PTPRN2* (hypermethylated in blood, hypomethylated in adipose tissue) were reported in more than one study. We found reports of hypermethylation of these genes that were associated with decreased gene expression, and vice versa. We also report findings from studies done on monozygotic twins. Various traits that can affect T2DM such as sex, glucose levels, BMI and ethnicity were also taken into consideration.

As there were multiple methods that were used to measure DNA methylation, internal and external validation of these results is also reported. Finally, animal model studies that have reported differential DNA methylation of the genes that were found to be differentially methylated in human studies were looked at to get an understanding of the likely mechanisms linking epigenetic dysregulation of these genes in T2DM to its pathophysiology.

Although the majority of the top differentially methylated genes are well known, other more recent genes reported here should be investigated further to understand their role in pathogenesis of T2DM.

## Data availability statement

All relevant data are presented as tables and/or figures.

### Supplementary Information

Additional file 1 Search strategy for the systematic review of DNA methylation association with T2DM

Additional file 2 Qualitative assessment of research articles included in the review based on the New Castle Ottawa Scale (NOS)

## Abbreviations

*ABCG1*: ATP-Binding Cassette Subfamily G Member 1
*ANO8*: Anoctamin 8
*B3GNT7*: Beta 1,3-N-Acetylglucosaminyltransferase 7
*C1orf52*: Chromosome 1 Open Reading Frame 52
*C7orf29*: Chromosome 7 Open Reading Frame 29
*COL21A1*: Collagen Type XXI Alpha 1
*CYB561D1*: Cytochrome B561 Family Member D1
CXXC4: CXXC Finger Protein 4
*FTO*: Alpha-Ketoglutarate Dependent Dioxygenase
*GLP1R*: Glucagon Like Peptide 1 Receptor
*Gpx6*: Glutathione Peroxidase 6
*HNF4A*: Hepatocyte Nuclear Factor 4 Alpha
*HOOK2*: Hook Microtubule Tethering Protein 2
*IGFBP2*: Insulin-Like Growth Factor-Binding Protein 2
*IL23Ap19*: Interleukin-23 Subunit Alpha
*KCNQ1*: Potassium Voltage-Gated Channel Subfamily Q Member 1
*L1TD1*: LINE1 Type Transposase Domain Containing 1
*LOXL2*: Lysyl Oxidase Homolog 2
*MALT1*: Mucosa-Associated Lymphoid Tissue Lymphoma Translocation Protein 1
*MFSD1*: Major Facilitator Superfamily Domain Containing 1
*MSI2*: Musashi RNA-Binding Protein 2
*OPTN*: Optineurin
*PDGFA*: Platelet Derived Growth Factor Subunit A
*PDX1*: Pancreatic and Duodenal Homeobox 1
*PER2*: Period Circadian Regulator 2
*PHOSPHO1*: Phosphoethanolamine/Phosphocholine Phosphatase 1
*PPARGC1A*: Peroxisome Proliferator-Activated Receptor Gamma Coactivator 1-Alpha
*PROC*: Protein C, Inactivator Of Coagulation Factors Va And VIIIa
*PTBP1*: Polypyrimidine Tract-Binding Protein 1
*PTPRN2*: Protein Tyrosine Phosphatase Receptor Type N2
*RIPK4*: Receptor Interacting Serine/Threonine Kinase 4
*S100A4*: S100 Calcium-Binding Protein A4
*SAMD12*: Sterile Alpha Motif Domain Containing 12
*SLC145*: Solute Carrier Family 1 Member 5
*SLC22A1*: Solute Carrier Family 22 Member 1
*SLC22A3*: Solute Carrier Family 22 Member 3
*SLC30A8*: Solute Carrier Family 30 Member 8
*SREBF1*: Sterol Regulatory Element-Binding Transcription Factor 1
*SOCS3*: Suppressor Of Cytokine Signaling 3
*TCF7L2*: Transcription Factor 7-Like 2
*TXNIP*: Thioredoxin-Interacting Protein
