# Supplementary material for: DNA methylation and type 2 diabetes: a systematic review
Source: Clin Epigenetics. 2024 May 16;16:67. doi: 10.1186/s13148-024-01670-6 (PMC11100087; doi:10.1186/s13148-024-01670-6)
Supplement: Supplementary file 1 — Additional file 1 Search strategy for the systematic review of DNA methylation association with T2DM [file 13148_2024_1670_MOESM1_ESM.docx]

Supplementary table

**Supplementary table S1**. Search strategy for the systematic review of DNA methylation association with T2DM.

| **Database** | **Keywords** | **Results** |
| --- | --- | --- |
| PubMed | ("T2DM" OR "type 2 diabetes mellitus" OR "NIDDM" OR “diabetes mellitus type 2") AND ("DNA methylation" OR "5-methylcytosine" OR "CpG methylation" OR "epigenetics" OR "epigenomics") | 977 |
| Science Direct | ("T2DM" OR "type 2 diabetes mellitus" OR "NIDDM" OR "diabetes mellitus type 2") AND ("DNA methylation" OR "5-methylcytosine" OR "CpG methylation" OR "epigenetics" OR "epigenomics") | 4842 |
