# Supplementary material for: DNA methylation and type 2 diabetes: a systematic review
Source: Clin Epigenetics. 2024 May 16;16:67. doi: 10.1186/s13148-024-01670-6 (PMC11100087; doi:10.1186/s13148-024-01670-6)
Supplement: Supplementary file 2 — Additional file 2 Qualitative assessment of research articles included in the review based on the New Castle Ottawa Scale (NOS) [file 13148_2024_1670_MOESM2_ESM.docx]

**Supplementary Table 2**. Qualitative assessment of research articles included in the review based on the New Castle Ottawa Scale (NOS).

| **Author, year** | **Adequacy of case definition** | **Representativeness of cases** | **Selection of controls** | **Definition of Controls** | **Comparability of cases and controls on the basis of the design or analysis** | **Total score** | **Reference** |
| --- | --- | --- | --- | --- | --- | --- | --- |
| Nilsson, et al. 2014 | * Yes, with independent validation | * Consecutive or obviously representative series of cases | * Community controls | * No history of disease | ** Age and other factors | 6 | (36) |
| Rodríguez-Rodero, et al. 2017 | Yes, based on self-reports | * Consecutive or obviously representative series of cases | hospital controls | * No history of disease | ** Gastric bariatric surgery and glucose levels | 4 | (37) |
| Yuan, et al. 2014 | Yes, based on self-reports | * Consecutive or obviously representative series of cases | hospital controls | * No history of disease | **Age and zygosity | 4 | (53) |
| Kriebel, et al. 2016 | * Yes, with independent validation | * Consecutive or obviously representative series of cases | hospital controls | * No history of disease | ** Age, FBG, etc. | 5 | (18) |
| Wang, et al. 2018 | * Yes, with independent validation | * Consecutive or obviously representative series of cases | * Community controls | * No history of disease | ** Age, FBG, etc. | 6 | (38) |
| Volkmar M, et al. 2012 | * Yes, with independent validation | * Consecutive or obviously representative series of cases | hospital controls | * No history of disease | ** HbA1C and other factors | 5 | (3) |
| Jeon, et al. 2017 | * Yes, with independent validation | * Consecutive or obviously representative series of cases | hospital controls | * No history of disease | ** Age, FBG, etc. | 5 | (27) |
| Nilsson, et al. 2015 | * Yes, with independent validation | * Consecutive or obviously representative series of cases | hospital controls | * No history of disease | ** Age and other factors | 5 | (51) |
| Soriano-Tarraga, et al. 2016 | Yes, based on self-reports | * Consecutive or obviously representative series of cases | * Community controls | * No presence or history of disease | * Age, Sex, HbA1c, Blood pressure | 4 | (16) |
| Wang, et al. 2021 | * Yes, independently validated | No description | No description | No description | * Discordant twins | 2 | (17) |
| Meeks, et al. 2019 | * Yes, with independent validation | * Consecutive or obviously representative series of cases | * Community controls | * No presence or history of disease | * *Age, sex, HbA1c, BMI, FBS, HOMA-IR, HOMA-B, family history of diabetes, smoking habits, alcohol intake, WBC count | 6 | (20) |
| Ribel-Madsen, et al. 2012 | * Yes, with independent validation | * Consecutive or obviously representative series of cases | * Community controls | * No presence or history of disease | * *Age, Sex, HbA1c | 6 | (39) |
| Dayeh, et al. 2014 | Yes, record linkage | * All cases in a defined hospital | Hospital controls | * No presence or history of disease | ** Age, Sex, BMI, HbA1c, Glucose-stimulated insulin secretion | 4 | (28) |
| Hwang, et al. 2018 | * Yes, with independent validation | * Consecutive or obviously representative series of cases | * Community controls | * No presence or history of disease | Not specified | 4 | (93) |
| Toperoff, et al. 2012 | No description | * All cases in a defined catchment area | * Community controls | No description | ** Age, BMI, HbA1c | 4 | (58) |
| Zou, et al. 2013 | No description | Not stated | No description | * No presence or history of disease | No description | 1 | (94) |
| Abderrahmani, et al. 2018 | * Hospital records | * All cases in a defined hospital | Hospital controls | * No presence or history of disease | * *Age, Sex, BMI, CpG methylation | 5 | (50) |
| Chen, et al. 2016 | * Hospital records | * All cases in a defined hospital | No description | * No presence or history of disease | No description | 3 | (95) |
| Wittenbecher, et al. 2019 | * Yes, with independent validation | * Consecutive or obviously representative series of cases | * Community controls | * No presence or history of disease | ** Age, sex, fasting time before blood draw, time of blood sampling | 6 | (70) |
| Barres, et al. 2009 | No description | Not stated | No description | No description | ** Age, BMI, FBS, HbA1c, TG, VO_2_max | 2 | (52) |
| Kulkarni, et al. 2015 | No description | * Consecutive cases | * Community controls | No description | ** Age, sex | 4 | (19) |
| Volkov, 2017 | No description | No description | Hospital controls | No description | ** Age, sex | 2 | (59) |
| Chen, et al. 2019 | No description | No description | No description | No description | *Age | 1 | (57) |
| Chen, et al. 2020 | * Independently validated | * All cases in a defined hospital | * Community controls | * No presence or history of disease | ** Age, Sex, BMI, HbA1c, FBS | 6 | (56) |
| Davegardh, et al. 2021 | No description | No description | Hospital controls | * No presence or history of disease | ** Age, sex, BMI | 3 | (96) |
| Tsai, et al. 2022 | * Yes, independently validated | *All cases in a defined catchment area | *Community controls | *No presence or history of disease | **BMI, Age, HbA1c, FBS | 6 | (23) |
| Xiang, et al. 2021 | * Yes, independently validated | No description | No description | No description | * Generalized estimating equations (GEE) were applied to adjust for the relatedness between co-twins with T2D status as dichotomous dependent variable | 2 | (21) |
| Dawes, et al. 2022 | * Yes, independently validated | * All cases in a defined catchment area | *Community controls | * No history of disease | * Blood glucose levels | 5 | (22) |
| Florath, et al. 2016 | * Hospital records | * All cases in a defined catchment area | *Community controls | * No history of disease | * Blood glucose levels | 5 | (25) |
| Khamis, et al. 2022 | * Yes, independently validated | * Consecutive or obviously representative series of cases | *Community controls | *No presence or history of disease | * Blood glucose levels | 5 | (24) |
| Whytock, et al. 2023 | * Yes, independently validated | No description | No description | No description | * Blood glucose levels | 2 | (97) |
| Andersen, et al. 2019 | * Yes, independently validated | * All cases in a defined hospital | Hospital controls | * No presence or history of disease | Not specified | 3 | (40) |

FBS: fasting blood glucose; HbA1c: glycated hemoglobin; HOMA-IR: homeostatic model assessment for insulin resistance; HOMA-B: homeostatic model assessment of beta cell function; BMI: body mass index; WBC: white blood cells; HDL: high density lipoprotein; LDL: low density lipoprotein; TG: triglyceride; VO2max: maximal oxygen consumption; PP: pulse pressure; FPG: fasting plasma glucose; TC: total cholesterol.
